# MiR-520d-5p modulates chondrogenesis and chondrocyte metabolism through targeting HDAC1

**DOI:** 10.18632/aging.103831

**Published:** 2020-09-20

**Authors:** Jiajia Lu, Zhibin Zhou, Bin Sun, Bin Han, Qiang Fu, Yaguang Han, Wang Yuan, Zeng Xu, Aimin Chen

**Affiliations:** 1 Department of Orthopedics and Trauma Surgery, Changzheng Hospital, Shanghai, P. R. of China; 2 Department of Medicinal and Materials, General Hospital of Northern Theater Command, Shenyang, P. R. of China

**Keywords:** osteoarthritis, chondrogenesis, chondrocytes metabolism, human mesenchymal stem cells, miR-520d-5p

## Abstract

MicroRNAs (miRNAs) play an essential role in the chondrogenesis and the progression of osteoarthritis (OA). This study aimed to determine miRNAs associated with chondrogenesis of human mesenchymal stem cells (hMSCs) and chondrocyte metabolism. MiRNAs were screened in hMSCs during chondrogenesis by RNA-seq and qRT-PCR. MiRNA expression was determined in primary human chondrocytes (PHCs), and degraded cartilage samples. MiRNA mimics and inhibitors were transfected to cells to determine the effect of miRNA. Bioinformatic analysis and luciferase reporter assays were applied to determine the target gene of miRNA. The results demonstrated that miR-520d-5p was increased in hMSCs chondrogenesis. The overexpression and knockdown of miR-520d-5p promoted and inhibited chondrogenesis, and regulated chondrocyte metabolism. Histone deacetylase 1 (HDAC1) was decreased in hMSCs chondrogenesis, and HDAC1 was a targeting gene of miR-520d-5p. CI994, HDAC1 inhibitor, elevated cartilage-specific gene expressions and promoted hMSCs chondrogenesis. In IL-1β-treated PHCs, CI994 promoted AGGRECAN expression and suppressed MMP-13 expression, abolishing the effect of IL-1β on PHCs. Taken together, these results suggest that miR-520d-5p promotes hMSCs chondrogenesis and regulates chondrocyte metabolism through targeting HDAC1. This study provides novel understanding of the molecular mechanism of OA progression.

## INTRODUCTION

Osteoarthritis (OA) is the most widespread joint degenerative disease around the world and a leading cause of pain and severe impairment of mobility in adults [1]. It is estimated that OA influences around 18% of females and 10% of males over 60 years old, leading to a substantial socioeconomic burden [2]. OA is characterized by degraded articular cartilage, subchondral bone thickening, synovial inflammation, osteophyte formation, and degeneration of ligaments [3]. Growing studies reveal that the etiology of OA is a complicated multi-factorial mechanism related to heredity, aging, joint injury, as well as obesity [4]. To date, the detailed mechanisms underlying OA progression remain to be thoroughly investigated, and there are no effective interventions to cure degraded cartilage or slow OA progression.

As a primary pathological manifestation, cartilage degeneration takes place in the presence of mechanical damages or low-grade local or systemic inflammatory responses related to obesity, trauma, genetic predisposition, and metabolic syndrome, which are the main risk factors for the initiation and progression of OA [5, 6]. In contrast, chondrogenesis is a process of the generation of chondrocytes that occurs both embryogenesis and adulthood, resulting in the formation of cartilage [7]. Chondrocytes originate from the chondrogenic differentiation of mesenchymal stem cells (MSCs) [8], in which a number of cytokines and transcription factors are associated with the chondrocyte differentiation, including Runx protein family (Runx1, Runx2, and Runx3) [9] and Sox family (Sox5, Sox6, and Sox9) [10]. Beyond that, chondrocyte-specific enhancer elements, such as COL2A1, COL9A1, COL10A2, and AGGRECAN, interact with transcription factors to impact cartilage-specific gene expressions, indicating that the chondrogenesis is involved in multiple factors and signaling pathways [11]. Therefore, much attention so far is being drawn toward investigating the cellular and molecular mechanisms underlying the cartilage degeneration and chondrogenesis in OA.

In the past decade, microRNAs (miRNAs), a class of non-coding short RNAs of 18-22 nucleotides [12], are critical regulatory factors for gene expression at the post-transcriptional level, in which miRNAs can inhibit translation through directly binding to 3'UTR of target mRNA and promote mRNA degradation [13]. Numerous studies suggest that miRNAs participate in various cellular processes, such as proliferation, inflammation, stress response, apoptosis, and migration [14]. It has been demonstrated that miRNAs play an essential role in the regulation of cartilage degeneration and chondrogenesis [15, 16]. For example, the expression of miR-194 decreases during the chondrogenesis of human adipose-derived stem cells (hASCs), and the downregulation of miR-194 promotes the expression of SOX5, leading to enhanced chondrogenic differentiation [17]. The upregulation of miR-365 in the pre-hypertrophic zone plays a positive role in chondrocyte proliferation by targeting histone deacetylase 4 (HDAC4) [18].

Histone deacetylases (HDACs) is an essential enzyme family associated with the modulation of histone acetylation [19], an important mechanism modulating the gene expression at the transcriptional level [20]. Currently, HDACs are classified into four families (class I, II, III, and IV), according to their function, structure, distribution, as well as expression pattern [21–23], of which class I, including HDAC1, 2, 3, and 8, has been reported to be essential for the progression of osteoarthritis and chondrogenesis [24]. HDAC1, in particular, plays a vital role in murine development while it is required for the formation of craniofacial cartilage and pectoral fins in zebrafish [25]. Higher expressions of HDAC1 and HDAC2 are found in the chondrocytes of diseased cartilages of patients with OA, which inhibit the expressions of matrix proteins, such as AGGRECAN and COL2A1 [26]. In addition, HDAC1 promotes TGF-β1-mediated chondrogenesis through downregulating canonical Wnt signaling pathway [27].

In this study, RNA-seq analysis and functional experiments revealed that miR-520d-5p was an essential miRNA associated with the chondrogenic differentiation of human mesenchymal stem cells (hMSCs) and chondrocyte metabolic activities. By bioinformatic prediction, we found that miR-520d-5p might directly target HDAC1. In addition, we demonstrated that miR-520d-5p plays an essential role in chondrogenesis and cartilage degradation by inhibiting HDAC1, thereby modulating the expressions of cartilage-specific genes.

## RESULTS

### Elevated expression of miR-520d-5p and decreased level of HDAC1 in hMSCs chondrogenesis

**Figure 1:**
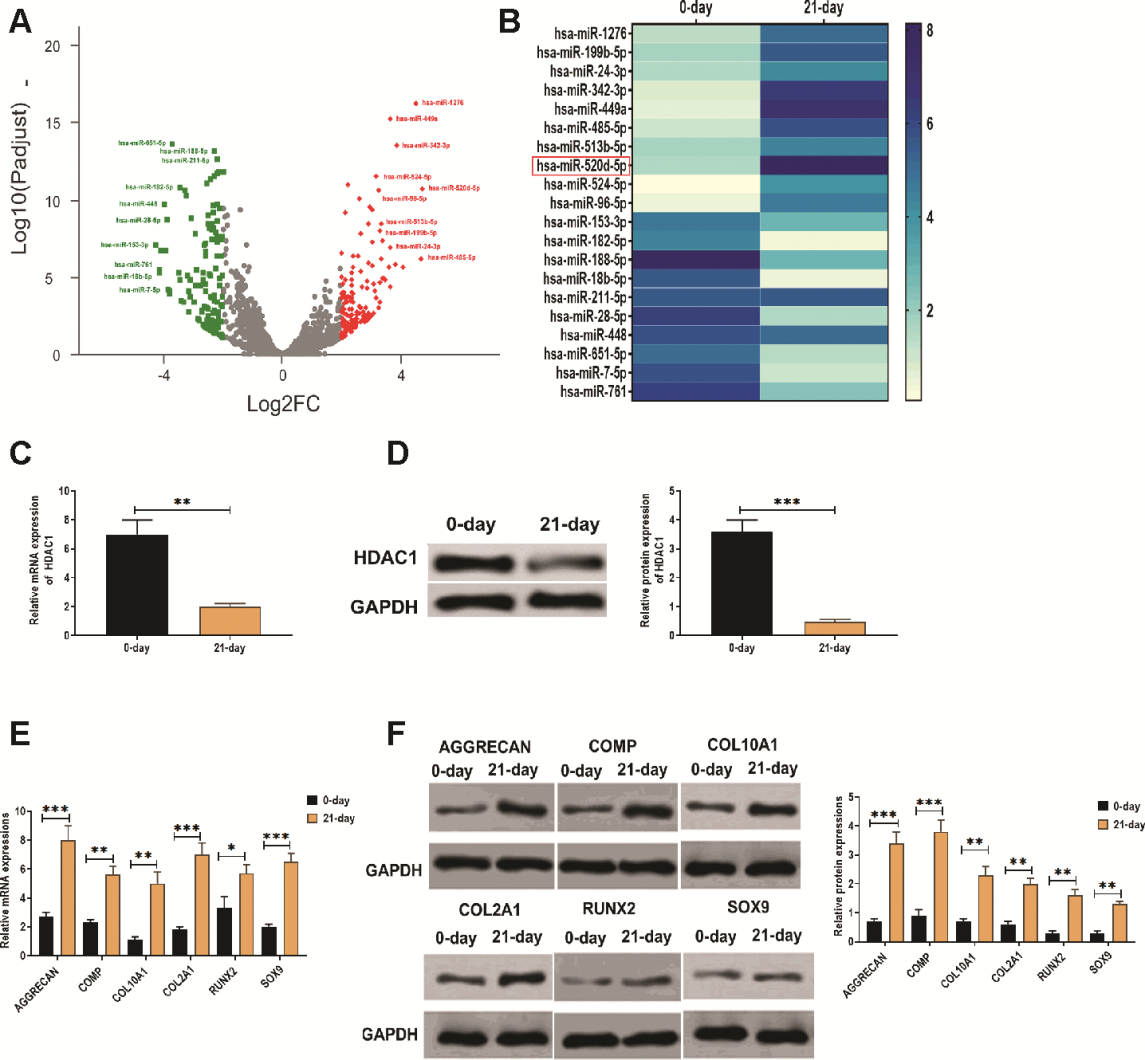
**Elevated expression of miR-520d-5p and decreased level of HDAC1 in the chondrogenesis of hMSCs.** (**A**) Volcano plot showing the differentially expressed miRNAs in hMSCs during chondrogenesis at 0 days and 21 days, as detected by RNA-seq assay. Red and green dots represent upregulated and downregulated miRNAs, respectively, according to fold change > 2 and *p* < 0.05. (**B**) Heatmap showing the expressions of differentially expressed miRNAs, as detected by qRT-PCR. (**C**) mRNA expression of HDAC1 in hMSCs at 0 days and 21 days of chondrogenesis. (**D**) Protein expression of HDAC1 in hMSCs at 0 days and 21 days of chondrogenesis. (**E**) mRNA expressions of the chondrogenic markers AGGRECAN, COMP, COL2A1, and SOX9 and the hypertrophic markers COL10A1 and RUNX2 at 0 days and 21 days of chondrogenesis. (**F**) Protein expressions of the chondrogenic markers AGGRECAN, COMP, COL2A1, and SOX9 and the hypertrophic markers COL10A1 and RUNX2 at 0 days and 21 days of chondrogenesis. For each experiment, at least three replicates were available for the analysis. Data were expressed as mean ± standard deviation (SD). *P < 0.05; ** P < 0.01; *** P < 0.001.

By RNA-Seq analysis, a group of differentially expressed miRNAs was found in hMSCs that were induced to differentiate to chondrocytes at 0 days and 21 days (Figure 1A), of which miR-520d-5p was one of the upregulated miRNAs (Figure 1A). We then applied qRT-PCR to verify the results obtained from RNA-Seq assay, we found that miR-520-5p showed the highest increasing rate compared with other upregulated miRNAs in hMSCs (Figure 1B). These results suggested that miR-520d-5p might be an essential regulator in the chondrogenesis of hMSCs. Meanwhile, we also found that mRNA and protein expressions of HDAC1 was decreased in hMSCs at 21 days after chondrogenic induction (Figure 1C and 1D). In addition, both mRNA and protein expressions of chondrogenic markers AGGRECAN, COMP, COL2A1, and SOX9 and the hypertrophic markers COL10A1 and RUNX2 were elevated in hMSCs at 21 days after chondrogenic induction, compared with those at 0 days of the chondrogenesis (Figure 1E and 1F).

### MiR-520d-5p inhibited the expression of HDAC1 in chondrogenesis hMSCs

**Figure 2:**
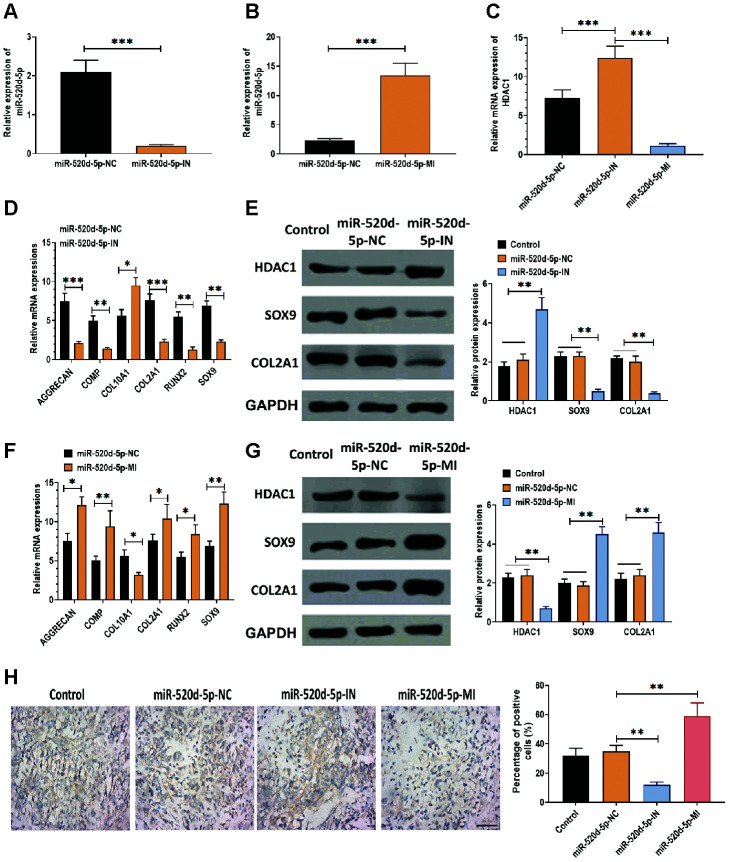
**miR-520d-5p affects the expression of HDAC1 in hMSCs during chondrogenesis.** In these experiments, hMSCs were transfected with miR-520d-5p inhibitor (miR-520d-5p-IN) or miR-520d-5p mimics (miR-520d-5p-MI) at 0 day and then were induced to differentiate into chondrocytes for 21 days. All experiments were conducted at 21 days after chondrogenic induction. (**A**, **B**) Expressions of miR-520d-5p in hMSCs. (**C**) mRNA expressions of HDAC1 in hMSCs. (**D**) mRNA expressions of the chondrogenic markers AGGRECAN, COMP, COL2A1, and SOX9 and the hypertrophic markers COL10A1 and RUNX2 in hMSCs transfected with miR-520d-5p-IN at 0 days of chondrogenesis. (**E**) Protein expressions of HDAC1, SOX9, and COL2A1 in hMSCs transfected with miR-520d-5p-IN at 0 days of chondrogenesis. (**F**) mRNA expressions of the chondrogenic markers AGGRECAN, COMP, COL2A1, and SOX9 and the hypertrophic markers COL10A1 and RUNX2 in hMSCs transfected with miR-520d-5p-MI at 0 days of chondrogenesis. (**G**) Protein expressions of HDAC1, SOX9, and COL2A1 in hMSCs transfected with miR-520d-5p-MI at 0 days of chondrogenesis. (**H**) Immunohistochemistry assay for collagen type II in hMSCs transfected with miR-520d-5p-IN or miR-520d-5p-MI at 0 days of chondrogenesis (16× magnification). Scale bar = 100 μm. For each experiment, at least three replicates were available for the analysis. Data were expressed as mean ± standard deviation (SD). *P < 0.05; ** P < 0.01; *** P < 0.001.

According to the observation that miR-520d-5p and HDAC1 displayed opposite expression patterns in hMSCs during chondrogenesis, we used miR-520d-5p inhibitors and mimics to knockdown and overexpress miR-520d-5p in hMSCs. The results suggested that the levels of miR-520d-5p were dramatically inhibited and enhanced in hMSCs transfected with miR-520d-5p inhibitors and miR-520d-5p mimics, respectively (Figure 2A and 2B). Also, we found that the mRNA expressions of HDAC1 were increased in hMSCs transfected with miR-520d-5p inhibitors while decreased in those treated with miR-520d-5p mimics (Figure 2C). In addition, the knockdown of miR-520d-5p was associated with the decreased mRNA expressions of AGGRECAN, COMP, COL2A1, SOX9, RUNX2, whereas increased expression of COL10A1 (Figure 2D). The opposite results were observed in hMSCs with overexpression of miR-520d-5p (Figure 2F). For protein expressions, miR-520d-5p inhibitors increased the expression of HDAC1 while suppressed the levels of SOX9 and COL2A1 (Figure 2E). In contrast, miR-520d-5p mimics inhibited the expression of HDAC1 while enhanced the expression of SOX9 and COL2A1 in hMSCs (Figure 2G). Furthermore, as shown in the immunohistochemistry assay for collagen type II, the knockdown of miR-520d-5p played a negative role in the chondrogenic differentiation of hMSCs, which was opposite to the effect of overexpression of miR-520d-5p on the chondrogenesis of hMSCs (Figure 2H). As such, these results indicate that there is an inverse correlation between miR-520d-5p and HDAC1 and that miR-520d-5p is associated with hMSCs chondrogenesis.

### Expressions of miR-520d-5p and HDAC1 in IL-1β-treated PHCs

**Figure 3:**
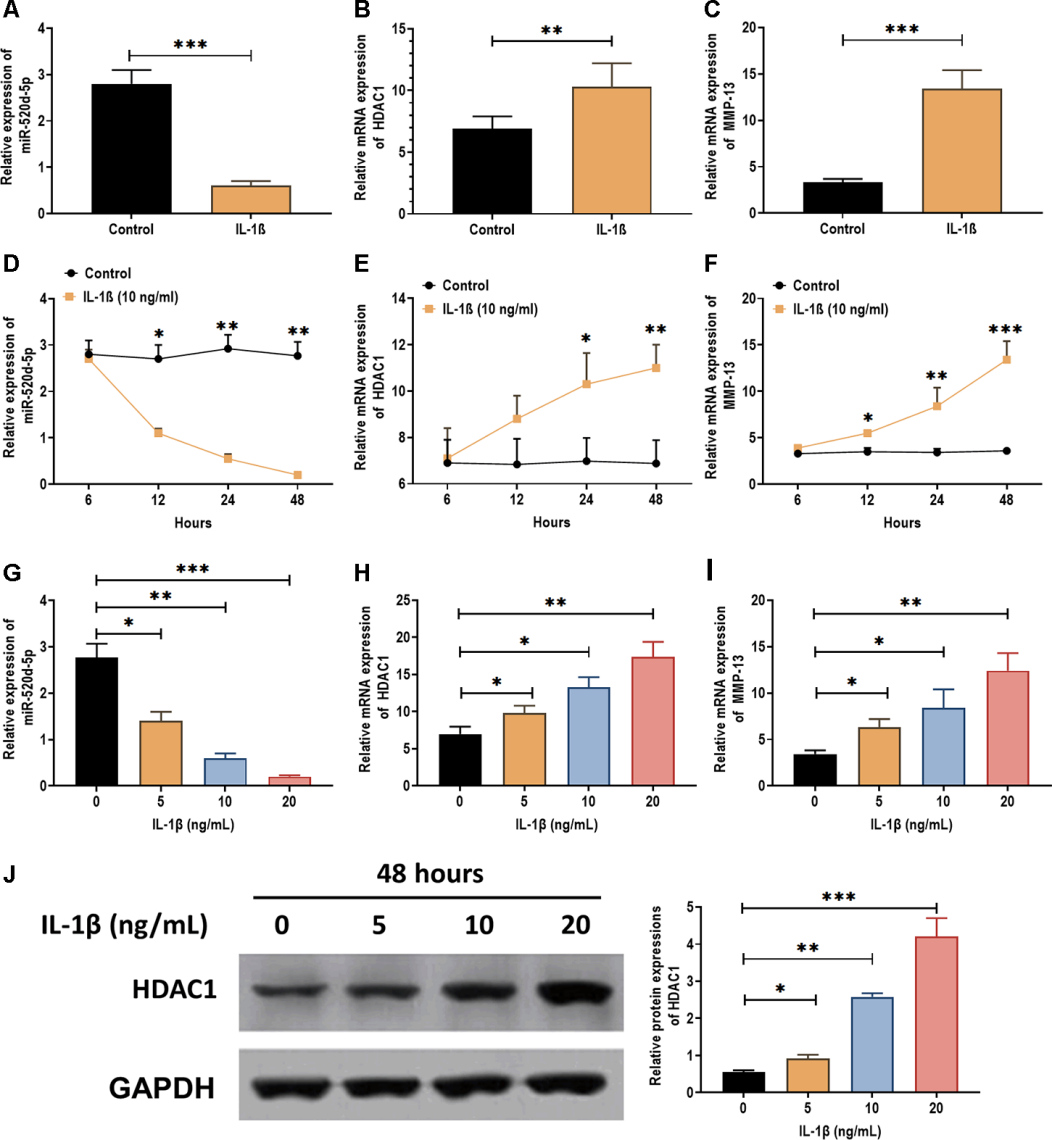
**Expressions of miR-520d-5p and HDAC1 in IL-1β-treated PHCs.** (**A**–**C**) Expressions of miR-520d-5p, HDAC1, and MMP-13 in IL-1β-treated PHCs at 24 hours post-IL-1β treatment (5 ng/mL), respectively. (**D**–**F**) Expressions of miR-520d-5p, HDAC1, and MMP-13 in IL-1β-treated PHCs at 6, 12, 24, and 48 hours post-IL-1β treatment (10 ng/mL), respectively. (**G**–**I**) Expressions of miR-520d-5p, HDAC1, and MMP-13 in PHCs treated with 0, 5, 10, 20 ng/mL IL-1β at 24 hours post-treatment, respectively. (**J**) Protein expression of HDAC1 in PHCs treated with 0, 5, 10, 20 ng/mL IL-1β at 48 hours post-treatment. For each experiment, at least three replicates were available for the analysis. Data were expressed as mean ±standard deviation (SD). *P < 0.05; ** P < 0.01; *** P < 0.001.

IL-1β has been demonstrated as a proinflammatory cytokine in OA cartilage degradation and plays an inhibitory role in the chondrogenesis [28, 29] and chondrocytes metabolism [30]. Thus, we attempted to determine the effect of IL-1β on the expression of miR-520d-5p. The results showed that IL-1β treatment (5 ng/mL) significantly decreased the expression of miR-520d-5p (Figure 3A) while increased the mRNA levels of HDAC1 and MMP-13 (Figure 3B and 3C). In addition, the effect of IL-1β on the expressions of miR-520d-5p, HDAC1, and MMP-13 exhibited a time- and dose-dependent manner (Figure 3D–3I). By western blotting assay, the protein expression of HDAC1 was increased by IL-1β treatments in a dose-dependent manner (Figure 3J). These results together demonstrate that IL-1β treatment exerts the opposite role in the expressions of miR-520d-5p and HDAC1, compared with those in chondrogenesis.

### miR-520d-5p promotes chondrocytes metabolism by targeting HDAC1

**Figure 4:**
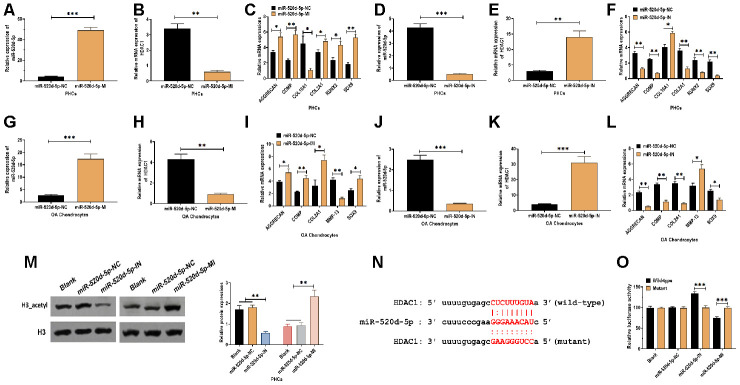
**miR-520d-5p regulates the chondrocyte metabolism through targeting HDAC1.** (**A**, **B**) Expressions of miR-520d-5p and HDAC1 in PHCs treated with miR-520d-5p negative control (miR-520d-5p-NC) or miR-520d-5p mimics at 24 hours post-treatment, respectively. (**C**) mRNA expressions of the chondrogenic markers AGGRECAN, COMP, COL2A1, and SOX9 and the hypertrophic markers COL10A1 and RUNX2 in PHCs treated with miR-520d-5p negative control (miR-520d-5p-NC) or miR-520d-5p mimics at 24 hours post-treatment. (**D**, **E**) Expressions of miR-520d-5p and HDAC1 in PHCs treated with miR-520d-5p negative control (miR-520d-5p-NC) or miR-520d-5p inhibitors at 24 hours post-treatment, respectively. (**F**) mRNA expressions of the chondrogenic markers AGGRECAN, COMP, COL2A1, and SOX9 and the hypertrophic markers COL10A1 and RUNX2 in PHCs treated with miR-520d-5p negative control (miR-520d-5p-NC) or miR-520d-5p inhibitors at 24 hours post-treatment. (**G**–**H**) Expressions of miR-520d-5p and HDAC1 in OA chondrocytes treated with miR-520d-5p negative control (miR-520d-5p-NC) or miR-520d-5p mimics at 24 hours post-treatment, respectively. (**I**) mRNA expressions of the chondrogenic markers AGGRECAN, COMP, COL2A1, MMP-13, and SOX9 in OA chondrocytes treated with miR-520d-5p negative control (miR-520d-5p-NC) or miR-520d-5p mimics at 24 hours post-treatment. (**J**, **K**) Expressions of miR-520d-5p and HDAC1 in OA chondrocytes treated with miR-520d-5p negative control (miR-520d-5p-NC) or miR-520d-5p inhibitors at 24 hours post-treatment, respectively. (**L**) mRNA expressions of the chondrogenic markers AGGRECAN, COMP, COL2A1, MMP-13, and SOX9 in OA chondrocytes treated with miR-520d-5p negative control (miR-520d-5p-NC) or miR-520d-5p inhibitors at 24 hours post-treatment. (**M**) Acetylation of histone H3 in PHCs treated with miR-520d-5p negative control (miR-520d-5p-NC), miR-520d-5p inhibitors, or miR-520d-5p mimics at 48 hours post-treatment, respectively. (**N**) The putative or mutated miR-520d-5p binding site at 3'UTR of HDAC1. (**O**) Luciferase reporter assay in PHCs with wild-type or mutant 3’UTR in the presence of miR-520d-5p negative control (miR-520d-5p-NC), miR-520d-5p inhibitors, or miR-520d-5p mimics. For each experiment, at least three replicates were available for the analysis. Data were expressed as mean ± standard deviation (SD). *P < 0.05; ** P < 0.01; *** P < 0.001.

To further determine the correlation between miR-520d-5p and HDAC1 in chondrocytes, PHCs, and OA chondrocytes were transfected with miR-520d-5p inhibitors or miR-520d-5p mimics (Figure 4A, 4D, 4G, and 4J). In PHCs, the overexpression of miR-520d-5p dramatically inhibited the mRNA expression of HDAC1 and COL10A1 while enhanced the mRNA levels of AGGRECAN, COMP, COL2A1, RUNX2, and SOX9 (Figure 4B and 4C). Meanwhile, the knockdown of miR-520d-5p significantly increased the mRNA expressions of HDAC1 and COL10A1, whereas decreased the expressions of AGGRECAN, COMP, COL2A1, RUNX2, and SOX9 (Figure 4E and 4F). In OA chondrocytes, forced expression of miR-520d-5p suppressed the mRNA expressions of HDAC1 and MMP-13 while promoted the mRNA expressions of AGGRECAN, COMP, COL2A1, and SOX9 (Figure 4H and 4I). Also, the suppression of miR-520d-5p showed the opposite role, compared with those of the overexpression of miR-520d-5p (Figure 4K and 4L). By western blotting assay, the knockdown of miR-520d-5p was associated with decreased protein expression of acetylated histone H3, whereas the overexpression of miR-520d-5p elevated the level of acetylation of histone H3 (Figure 4M). By predicting on bioinformatics programs, including miRanda [31], TargetScan [32] as well as miRWalk [33], the results revealed a putative miR-520d-5p binding site in 3'UTR of HDAC1 (Figure 4N). Then, luciferase reporter plasmids containing wild-type or mutant miR-520d-5p binding site were transfected to PHCs. We found that the luciferase activity was increased in PHCs transfected with plasmids containing wild-type miR-520d-5p binding site in the presence of miR-520d-5p inhibitor while was decreased in response to miR-520d-5p mimics (Figure 4O). Collectively, the results revealed that miR-520d-5p regulates chondrocytes metabolism by directly targeting HDAC1.

### CI994 promotes chondrogenic differentiation of hMSCs

**Figure 5:**
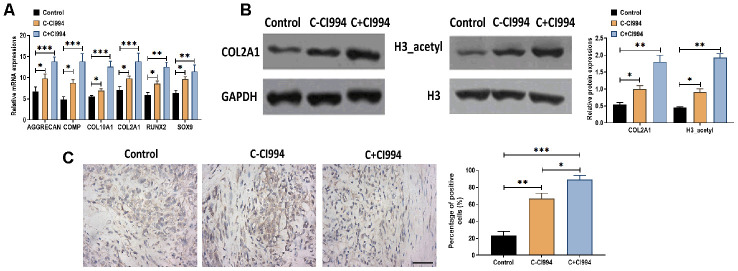
**CI994 promotes chondrogenic differentiation of hMSCs.** (**A**) mRNA expressions of the chondrogenic markers AGGRECAN, COMP, COL2A1, and SOX9 and the hypertrophic markers COL10A1 and RUNX2 in hMSCs treated with negative control (control), chondrogenic culture without CI994 (C-CI994), and chondrogenic culture with CI994 (C+CI994) (100 nM) at 21 days post-treatment. (**B**) Protein expression of COL2A1 and acetylation of histone H3 (H3_acetyl) in hMSCs treated with negative control (control), chondrogenic culture without CI994 (C-CI994), and chondrogenic culture with CI994 (C+CI994) (100 nM) at 21 days post-treatment. (**C**) Immunohistochemistry assay for collagen type II in hMSCs treated with negative control (control), chondrogenic culture without CI994 (C-CI994), and chondrogenic culture with CI994 (C+CI994) (100 nM) at 21 days post-treatment (16× magnification). Scale bar = 100 μm. For each experiment, at least three replicates were available for the analysis. Data were expressed as mean ± standard deviation (SD). *P < 0.05; ** P < 0.01; *** P < 0.001.

As an HDAC1 inhibitor [34], we applied CI994 (tacedinaline) in the chondrogenic medium to determine the role of CI994 in the chondrogenic differentiation of hMSCs. Compared with the chondrogenic medium without CI994, the treatment of CI994 (100 nM) increased the mRNA expressions of AGGRECAN, COMP, COL2A1, COL10A1, RUNX2, and SOX9 in hMSCs (Figure 5A). Meanwhile, the protein expressions of COL2A1 and acetylated histone H3 were also increased by CI994 treatment supplemented in the chondrogenic medium (Figure 5B). Immunohistochemistry assay revealed that CI994 treatment was associated with more Collagen type II-positive cells compared with those in the chondrogenic medium without CI994 (Figure 5C).

### CI994 reverses the effect of IL-1β on PHCs

**Figure 6:**
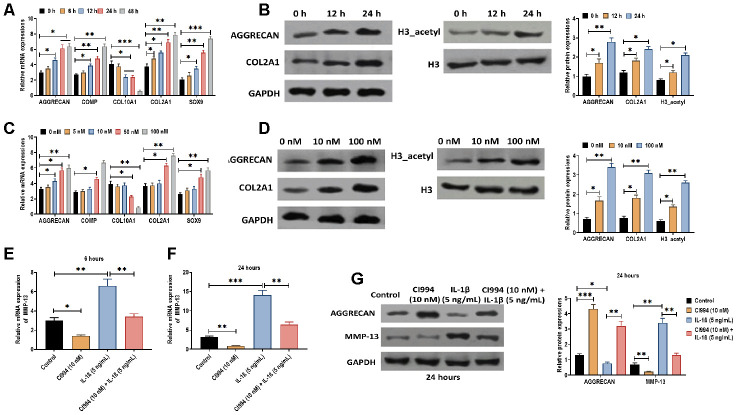
**CI994 reverses the effect of IL-1β in PHCs.** (**A**) mRNA expressions of the chondrogenic markers AGGRECAN, COMP, COL2A1, and SOX9 and the hypertrophic marker COL10A1 in PHCs treated with CI994 (10 nM) at 0, 6, 12, 24, and 48 hours post-treatment. (**B**) Protein expressions of AGGRECAN and COL2A1 and acetylation of histone H3 (H3_acetyl) in PHCs treated with CI994 (10 nM) at 0, 12, and 48 hours post-treatment. (**C**) mRNA expressions of the chondrogenic markers AGGRECAN, COMP, COL2A1, and SOX9 and the hypertrophic marker COL10A1 in PHCs treated with 0, 5, 10, 50, and 100 nM CI994 at 12 hours post-treatment. (**D**) Protein expressions of AGGRECAN and COL2A1 and acetylation of histone H3 (H3_acetyl) in PHCs treated with 0, 10, and 100 nM CI994 at 12 hours post-treatment. (**E**, **F**) mRNA expression of MMP-13 in PHCs treated with CI994 (10 nM), IL-1β (5 ng/mL), or the combination of CI994 (10 nM) and IL-1β (5 ng/mL) at 6 and 24 hours post-treatment, respectively. (**G**) mRNA expressions of AGGRECAN and MMP-13 in PHCs treated with CI994 (10 nM), IL-1β (5 ng/mL), or CI994 (10 nM) plus IL-1β (5 ng/mL) at 24 hours post-treatment. For each experiment, at least three replicates were available for the analysis. Data were expressed as mean ± standard deviation (SD). *P < 0.05; ** P < 0.01; *** P < 0.001.

Next, we determined the effect of CI994 on PHCs. We found that CI994 treatment increased the mRNA expressions of AGGRECAN, COMP, COL2A1, and SOX9 while decreased the mRNA level of COL10A1 in time- and dose-dependent manner (Figure 6A and 6C). Also, the protein expressions of AGGRECAN, COL2A1, and acetylated histone H3 were increased by CI994 treatment in time- and dose-dependent manner (Figure 6B and 6D). Furthermore, based on the observations mentioned above, we uncovered that the effect of IL-1β on PHCs was reversed by CI994 treatment, in which the increased expression of MMP-13 was inhibited by CI994 treatment at 8 and 24 hours post-treatment (Figure 6E and 6F). The similar effect of CI994 treatment was also observed in the protein expressions of AGGRECAN and MMP-13 (Figure 6G). Together, the results reveal that CI994 can reverse the effect of IL-1β in PHCs.

### Knockdown of HDAC1 attenuates the effect of the downregulation of miR-520d-5p in PHCs

**Figure 7:**
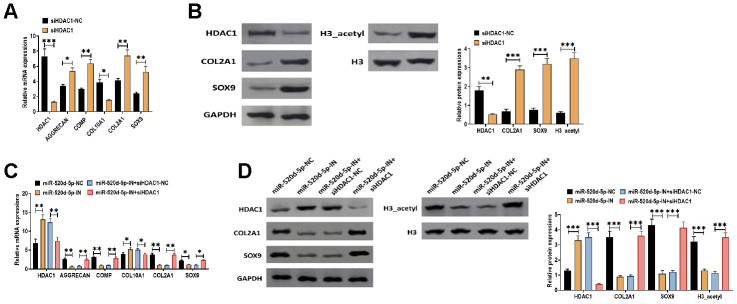
**Knockdown of HDAC1 attenuates the effect of the downregulation of miR-520d-5p in PHCs.** (**A**) mRNA expressions of HDAC1, the chondrogenic markers AGGRECAN, COMP, COL2A1, and SOX9 and the hypertrophic markers COL10A1 in PHCs treated with HDAC1 siRNA negative control (siHDAC1-NC) and HDAC1 siRNA (siHDAC1). (**B**) Protein expressions of HDAC1, COL2A1, and SOX9 and acetylation of histone H3 (H3_acetyl) in PHCs treated with HDAC1 siRNA negative control (siHDAC1-NC) and HDAC1 siRNA (siHDAC1). (**C**) mRNA expressions of HDAC1, the chondrogenic markers AGGRECAN, COMP, COL2A1, and SOX9 and the hypertrophic markers COL10A1 in PHCs treated with miR-520d-5p negative control (miR-520d-5p-NC), miR-520d-5p inhibitors (miR-520d-5p-IN), miR-520d-5p inhibitors (miR-520d-5p-IN) plus siRNA negative control (siHDAC1-NC), or miR-520d-5p inhibitors (miR-520d-5p-IN) plus HDAC1 siRNA (siHDAC1). (**D**) Protein expressions of HDAC1, COL2A1, and SOX9 and acetylation of histone H3 (H3_acetyl) in PHCs treated with miR-520d-5p negative control (miR-520d-5p-NC), miR-520d-5p inhibitors (miR-520d-5p-IN), miR-520d-5p inhibitors (miR-520d-5p-IN) plus siRNA negative control (siHDAC1-NC), or miR-520d-5p inhibitors (miR-520d-5p-IN) plus HDAC1 siRNA (siHDAC1). For each experiment, at least three replicates were available for the analysis. Data were expressed as mean ± standard deviation (SD). *P < 0.05; ** P < 0.01; *** P < 0.001.

To further determine the effect of HDAC1 in the chondrocytes metabolism, we applied HDAC1 siRNA to knockdown the expression of HDAC1 in PHCs. The results suggested that the knockdown of HDAC1 was associated with increased mRNA expressions of AGGRECAN, COMP, COL2A1, and SOX9 while decreased mRNA level of COL10A1 (Figure 7A). In addition, the inhibition of HDAC1 elevated the protein expressions of COL2A1, SOX9, and acetylated histone H3 (Figure 7B). Furthermore, we found that the effect of miR-520d-5p inhibitor was reversed by the knockdown of HDAC1, in which the decreased mRNA expressions of AGGRECAN, COMP, COL2A1, and SOX9 induced by miR-520d-5p inhibitor were reversed in the presence of HDAC1 siRNA (Figure 7C). Similar changing patterns were also found in the protein expressions of COL2A1, SOX9, and acetylated histone H3 (Figure 7D). Therefore, these results collectively suggested that the effect of the downregulation of miR-520d-5p can be reversed by the knockdown of HDAC1, further indicating the functional interaction between miR-520d-5p and HDAC1 in chondrocytes metabolism.

### Expressions of miR-520d-5p and HDAC1 in the degraded cartilage

**Figure 8:**
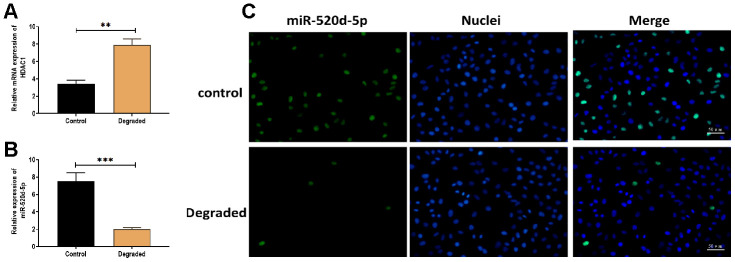
**Expressions of miR-520d-5p and HDAC1 in the degraded cartilage.** (**A**, **B**) Expression of miR-520d-5p and HDAC1 in non-degraded and degraded cartilages, respectively. (**C**) In situ hybridization for miR-520d-5p in non-degraded and degraded cartilages. MiR-520d-5p was labeled by green fluorescence, and the nuclei were labeled by blue fluorescence. Scalar bar = 50 μm. For each experiment, at least three replicates were available for the analysis. Data were expressed as mean ± standard deviation (SD). *P < 0.05; ** P < 0.01; *** P < 0.001.

To determine the effect of miR-520d-5p in the progression of OA, 21 pairs of non-degraded and degraded cartilage were used to detect the expressions of miR-520d-5p and HDAC1. The results revealed that the expression miR-520d-5p was lower while the level of HDAC1 was higher in degraded cartilage samples compared with those of non-degraded cartilages (Figure 8A and 8B). Also, the higher expression of miR-520d-5p was found in the fluorescent in situ hybridization assay (Figure 8C).

## DISCUSSION

OA is the most prevalent degenerative joint disease leading to pain and disability [35]. It has been forecast that total lived with disability (YLDs) due to OA rose from 0.84 million in 1990 to 1·97 million in 2017, and around 61.2 million individuals had suffered from OA in 2017 in China [36]. To date, in addition to pain management and surgical intervention, we still lack effective therapeutic treatment for OA. Therefore, there is an urgent need for investigating the underlying mechanism and alternative treatment for OA. In the present study, we reported that miR-520d-5p plays a positive role in hMSCs chondrogenesis through downregulating HDAC1 expression and modulates chondrocytes metabolism through impacting the expressions of cartilage-specific genes.

Many studies so far demonstrated that miR-520d-5p participates in diverse cellular processes. In human dermal fibroblast (NHDF) cells, miR-520d-5p repairs damages induced by ultraviolet B and restore damaged cells to normal senescent state through the survival of CD105-positive cells via c-Abl-ATR-BRCA1/p53 signaling pathways [37]. Given its anti-tumor effect, miR-520d-5p suppresses human glioma cell proliferation through targeting PTTG1 [38] and inhibits tumor metastasis and growth by binding to CTHRC1 in colorectal cancer [39]. In addition, the upregulation of miR-520d-5p is observed in the serum of patients with Parkinson's disease [40]. So far, however, there are no studies reporting the effect of miR-520d-5p on OA. To the best of our knowledge, we first demonstrated the role of miR-520d-5p in OA and chondrogenesis. In this study, we found that miR-520d-5p was upregulated in hMSCs at 21 days after chondrogenic induction. Also, miR-520d-5p regulated the expressions of cartilage-specific genes, such as AGGRECAN, COMP, COL2A1, and SOX9, which was also found in both PHCs and OA chondrocytes. In addition, we observed that IL-1β treatment, a proinflammatory cytokine in OA cartilage degradation [28, 29], inhibited the expression of miR-520d-5p in dose- and time-dependent manner. Furthermore, we further demonstrated that the level of miR-520d-5p was higher in the control cartilages, compared with those of degraded cartilages. Collectively, our results suggested that miR-520d-5p plays a positive role in the chondrogenic differentiation and chondrocyte metabolism.

As one of the functional mechanisms, miRNAs exert their effect through binding directly to the specific target genes [41]. To further determine the mechanism underlying the effect of miR-520d-5p on chondrogenesis, we applied bioinformatics analysis and luciferase reporter assay to demonstrated that HDAC1 was a target gene of miR-520d-5p, which can be used to reasonably interpret the opposite expression pattern between miR-520d-5p and HDAC1 in the chondrogenic differentiation and metabolism. As members of the class I HDAC family, HDAC1, HDAC2, and HDAC3 suppress chondrogenesis through downregulating cartilage-specific genes, including COL2A1, SOX9, and AGGRECAN, in human chondrocytes [26]. Also, HDAC1 is found to be involved in leukemia/lymphoma-related factor (LRF)-mediated suppressive effect on the chondrogenic differentiation [42]. Thus, these observations are consistent with the findings of this study, which suggests the negative effect of HDAC1 on chondrogenesis as well as cartilage-specific gene expression. In the present study, we also found that the effect of HDAC1 knockdown on cartilage-specific gene expressions was similar to those of overexpression of miR-520d-5p. This further demonstrated that the functional interaction between miR-520d-5p and HDAC1 in the chondrogenic differentiation. Meanwhile, these findings also suggested that miR-520d-5p might regulate the expressions of the cartilage-specific gene through inhibiting HDAC1 expression. In addition, we collected 21 pairs of non-degraded and degraded cartilage and measured the expressions of miR-520d-5p and HDAC1. The results showed that miR-520d-5p was downregulated, while HDAC1 was upregulated in degraded cartilages. This agrees with findings in vitro experiments in this study, further suggesting that miR-520d-5p can promote chondrogenesis, but HDAC1 can inhibit chondrogenic differentiation.

In conclusion, the results suggest that miR-520d-5p promotes hMSCs chondrogenesis and regulates chondrocyte metabolic activities through regulating the expressions of the cartilage-specific genes via targeting HDAC1. These observations provide a novel understanding of the mechanism underlying OA progression and insight into developing therapeutic treatments for OA.

## MATERIALS AND METHODS

### Ethics statement

All patients and subjects were informed before their inclusion, and written consent was given. The experimental protocols were approved by the Ethics Committee of Changzheng Hospital (Shanghai).

### Cell isolation and culture

Human mesenchymal stem cells (hMSCs) were isolated from the iliac crest bone marrow samples of three healthy volunteer donors (2 males and 1 female; 26-36 years old), as previously described [43]. Briefly, 10 mL bone marrow samples were diluted with 10 mL PBS buffer solution (Sigma-Aldrich, Shanghai, China). Then, hMSCs were fractionated on a Lymphoprep density-gradient through centrifugation at 500 g for 20 mins. The interfacial mononuclear cells were harvested, washed, and resuspended in DMEM (Dulbecco's Modified Eagle Medium) supplemented with 10% fetal bovine serum (FBS) (Gibco, NY, USA). Next, cells were seeded in a 25-cm^2^ flask (37 °C, 5% CO_2_) for 48 hours, and then nonadherent cells were removed by changing the new medium. When cell culture reached 80-90% confluence, cells were trypsinized by 0.25% trypsin (0.53mm EDTA), counted, and plated in a new cell culture dish contained DMEM (10% FBS, 100 μg/mL streptomycin, and 100 IU/mL penicillin). Human cartilage samples were collected from three subjects (1 male and 2 females; 38-48 years old) who underwent knee arthroplasty surgery between May 2017 and May 2018. Patients who were diagnosed as OA, immunological disorders, and tumors were excluded. Primary human chondrocytes (PHCs) were isolated from cartilage samples, as previously described [44, 45]. Briefly, cartilage samples were cut into small pieces (1 mm in diameter) and then digested with DMEM/F12 medium supplemented with 10% FBS, 100 μg/mL streptomycin, 100 IU/mL penicillin, and 0.4% Pronase (Gibco, NY, USA) for 90 mins. Then, the remaining cartilage tissue samples were transferred to second-digestion processes in DMEM/12 medium supplemented 100 μg/mL streptomycin, 100 IU/mL penicillin, 5% FBS (Gibco, NY, USA), and 0.025% Collagenase P (Sigma-Aldrich, Shanghai, China) for 7 hours at 37 °C on the plate stirrer. Afterward, chondrocytes were seed in a 25-cm^2^ flask with DMEM/F12 medium supplemented with 10% FBS, 100 μg/mL streptomycin, and 100 IU/mL penicillin (Gibco, NY, USA). Human OA chondrocytes were isolated from cartilage samples of three patients (2 males and 1 female; 31-38 years old), as described above. All three patients underwent lower limb amputation surgery between July 2017 and May 2018 and did not have the previous history of rheumatoid arthritis. OA was diagnosed based on the American College of Rheumatology criteria and corresponded to Kellgren–Lawrence OA grades III, and IV. For all cell culture procedures, culture media were replaced every three days, and all cells were cultured at 37 °C with 5% CO_2_. The cell culture reached 80-90% confluence, cells were detached by 0.05% trypsin/EDTA and then passaged in culture. hMSCs at passage 3 and PHCs at passage 1 were used to subsequent experiments.

### Human cartilage samples

Twenty-one pairs of non-degraded and degraded human articular cartilage samples were collected from twenty-one subjects who underwent knee arthroplasty surgery between August 2016 and May 2018 (10 males and 11 females; 47-64 years old). Subjects who were diagnosed as rheumatoid, inflammatory arthritis, immunological disorders, and tumors were excluded. Based on the Outerbridge classification scale [46], cartilage samples that were classified as grade 0 were defined as non-degraded cartilage, and cartilage samples that were classified as grade 2 and 3 were defined as degraded cartilage.

### Chondrogenic differentiation

Chondrogenic differentiation of hMSCs was performed as previously described [44]. Briefly, hMSCs (2 × 10^7^ cells) at passage 3 were harvested and resuspended in incomplete mesenchymal stem cell chondrogenic differentiation medium (Cyagen Biosciences, Suzhou, China; Cat. No. GUXMX-90041), including 96 ml basal medium, 10 μl dexamethasone, 100 μl sodium pyruvate, 100 μl proline, 300 μl ascorbic acid, 1 ml ITS, 1 ml transforming growth factor-β3. Droplets (12.5 μl) containing resuspended cells were then placed in each well of a 24-well plate. hMSCs were cultured at 37°C for 90 min to stimulate adherence to the plate, followed by the addition of 500 μL complete chondrogenic induction medium. Cell samples were harvested at different time points according to respective experimental design.

### qRT-PCR

Total RNAs from cells and cartilage samples were isolated using miRNeasy Mini kit (Qiagen, Hilden, Germany), following the manufacturer's instructions. The purity and concentration of RNAs were determined using Epoch Multi-Volume Spectrophotometer System (BioTek, VT, USA) according to the manufacturer's instructions. Reverse transcription was performed using PrimeScript® miRNA cDNA Synthesis kit (Takara Bio, Otsu, Japan) according to the manufacturer's instructions. Then, qRT-PCR reactions were performed using SYBR® Premix Ex Taq™ II (Takara Bio, Shiga, Japan) on the Bio-Rad IQ5 system platform (Bio-Rad Laboratories, Hercules, USA) according to the manufacturer's instructions. The primers used for target genes were summarized in Table 1. GAPDH and U6 RNA were used as the reference gene. Fold differences of target gene expressions were calculated using 2^-ΔΔCt^ method [47]. All samples were detected in triplicate.

**Table 1:** Primer sequences.

**Genes**	**Primers sequences**
**AGGRECAN_F**	5’- GATGTTCCCTGCAATTACCACCTC-3’
**AGGRECAN_R**	5'- TGATCTCATACCGGTCCTTCTTCTG-3'
**COMP_F**	5’- ACTCAGTTTCCCCACCATGTC -3’
**COMP_R**	5’- CAACGTCCAGCCTCAAGGTAA -3’
**COL10A1_F**	5’- CATAAAAGGCCCACTACCCAAC -3’
**COL10A1_R**	5’- ACCTTGCTCTCCTCTTACTGC -3’
**COL2A1_F**	5’- GCACCTGCAGAGACCTGAAAC -3’
**COL2A1_R**	5’- GCAAGTCTCGCCAGTCTCCA -3’
**SOX9_F**	5’- GGAGATGAAATCTGTTCTGGGAATG -3’
**SOX9_R**	5’- TTGAAGGTTAACTGCTGGTGTTCTG -3’
**RUNX2_F**	5’- ACCAATATGCAAGGCTTCACCAA -3’
**RUNX2_R**	5’- GCCTGTGTAACGCGAGCAGA -3’
**HDAC1_F**	5’- CCAAGTACCACAGCGATGAC -3’
**HDAC1_R**	5’- TGGACAGTCCTCACCAACG -3’
**MMP13_F**	5’- AAGACCCCAACCCTAAACATC -3’
**MMP13_R**	5’- AAAGATCATTGTTTCTCCTCGG -3’
**GAPDH_F**	5’- TGACGTGCCGCCTGGAGAAC -3’
**GAPDH_R**	5’- CCGGCATCGAAGGTGGAAGAG -3’
**U6_F**	5'- CTCGCTTCGGCAGCACA -3'
**U6_R**	5’- AACGCTTCACGAATTTGCGT -3’
**MiR-520d-5p**	5’- CTACAAAGGGAAGCCCTTTC -3’

### RNA-Seq

After checking the quantity and purity, total RNAs of hMSCs were used to prepare RNA library using TruSeq™ Small RNA Sample Prep Kits (Illumina, San Diego, USA) according to the manufacturer's instructions. Sequencing was performed on an Illumina Hiseq2500. Common RNA families (rRNA, tRNA, snRNA, snoRNA), adapter dimers, low complexity, and repeats were removed according to previously described [48]. Small RNA sequencing reads were aligned to miRbase build 20 using Bowtie software [49]. Normalized deep-sequencing counts in RPM (NOISeq) was applied to determine differential expression [50]. miRNAs whose expression are more than two-fold change with p< 0.05 were defined as differentially expressed miRNAs

### Western blotting

Total proteins were isolated from cells using lysis buffer. The amount of protein was determined using Bradford protein assay according to the manufacturer's instructions (Bio-Rad Laboratories, Hercules, USA). Protein samples (20 ug) were separated by SDS-PAGE and then transferred to PVDF membranes (Millipore, Bedford, USA). Membranes were incubated with primary antibodies overnight at 4 °C as follows: HDAC1 (1:1000), COL2A1 (1:2000), COL10A1 (1:1000), AGGRECAN (1:1000), COMP (1:1000), MMP-13 (1:1000) (Abcam, Shanghai, China); RUNX2 (1:2000), SOX9 (1:2000), total histone H3 (H3) (1: 5000), acetylated histone H3 (H3_acetyl)(1:2000) (Sigma-Aldrich, Shanghai, China); GAPDH (1:5000). The blots were incubated with secondary antibodies for 1 hour at room temperature. The blots were visualized by SuperSignal™ West Femto Maximum Sensitivity Substrate (Thermo Scientific™, Waltham, USA) according to the manufacturer's instructions. The intensity of brands was analyzed using Image J [51].

### Cell transfection and luciferase assay

hMSCs and PHCs (1 × 10^6^ cells) were transfected with antagomir-miR-520d-5p inhibitor (miR-520d-5p-IN) (100 nM), agomir-miR-520d-5p mimics (miR-520d-5p-MI) (100 nM), and miR-520d-5p negative control (miR-520d-5p-NC) (RiboBio, Guangzhou, China) [39]. PHCs (1 × 10^6^ cells) were transfected with HDAC1 siRNA (siHDAC1) and HDAC1 siRNA negative control (siHDAC1-NC) (Thermo Scientific™, Waltham, USA). Cell transfection procedures were performed using Lipofectamine® 3000 Transfection Reagent (Life Technologies, Carlsbad, USA) according to the manufacturer's instructions. For luciferase activity analysis, PHCs (1 × 10^6^ cells) were co-transfected with luciferase reporter plasmids containing wild-type or mutant miR-520d-5p binding site and miR-520d-5p-IN/ miR-520d-5p-MI (100 nM) using Lipofectamine® 3000 Transfection Reagent (Life Technologies, Carlsbad, USA) according to the manufacturer's instructions. Luciferase assay was performed using Dual-Luciferase® Reporter Assay System (Promega, Madison, USA) according to the manufacturer's instructions.

### Immunohistochemistry and Alcian blue staining

Immunohistochemistry assay was performed to determine the protein expressions of collagen type II, SOX9, and HDAC1, as previously described [52]. Meanwhile, samples were fixed with 4% paraformaldehyde and then embedded in paraffin. Processed samples were sectioned to 5 μm sections and stained with 1% Alcian blue 8GX for 30 min (Amresco, Solon, USA).

### *In situ* hybridization

Fluorescent in situ hybridization was performed to detect miR-520d-5p expression, as previously described [53]. The fluorescent probe for miR-520d-5p was obtained from Tocris Bioscience (Bristol, United Kingdom). Immunoreactivity was determined using Image-Pro Plus 6.0 analysis software.

### Statistical analysis

Data were expressed as mean ± standard deviation (SD). Statistical comparisons between groups were performed using Student's t-test or analysis of variance. Statistical analysis was performed using SPSS 13.0 (SPSS, Inc., Chicago, USA). P < 0.05 was defined as statistical significance. For each experiment, at least three replicates were available for the analysis.
